# When a Cure Becomes a Curse: The Complex Clinical Scenario Involving Amiodarone Therapy and BRASH (Bradycardia, Renal failure, Atrioventricular Nodal Blockade, Shock, and Hyperkalemia) Syndrome

**DOI:** 10.7759/cureus.38622

**Published:** 2023-05-06

**Authors:** Prabin Phuyal, Vishali Moond, Jesus A Catahay, Mario Caldararo, Keval V Patel

**Affiliations:** 1 Department of Internal Medicine, Saint Peter’s University Hospital/Rutgers Robert Wood Johnson Medical School, New Brunswick, USA; 2 Department of Pulmonary and Critical Care Medicine, Saint Peter’s University Hospital/Rutgers Robert Wood Johnson Medical School, New Brunswick, USA; 3 Cardiology, Saint Peter’s University Hospital/Rutgers Robert Wood Johnson Medical School, New Brunswick, USA

**Keywords:** amiodarone, brash syndrome, hyperkalemia, shock, acute renal failure, bradycardia

## Abstract

BRASH [bradycardia, renal failure, atrioventricular (AV) nodal blockade, shock, and hyperkalemia] syndrome is a recently recognized clinical condition that is rare but can be potentially life-threatening. Its pathogenesis is characterized by a self-perpetuating cycle of bradycardia that is potentiated by the concomitant occurrence of medication use, hyperkalemia, and renal failure. AV nodal blocking agents are commonly implicated in BRASH syndrome. We report a case of a 97-year-old female patient with a medical history of heart failure with preserved ejection fraction, atrial fibrillation, hypertension, hyperlipidemia, and hypothyroidism who presented to the emergency department with a one-day history of diarrhea and vomiting. Upon presentation, the patient was hypotensive, bradycardic, and had severe hyperkalemia, acute renal failure, and anion gap metabolic acidosis, raising concern for BRASH syndrome. The treatment of each component of BRASH syndrome resulted in the resolution of the symptoms. The association of BRASH syndrome with amiodarone, the only AV nodal blocking agent in this particular case, is not commonly reported.

## Introduction

BRASH [bradycardia, renal failure, atrioventricular (AV) nodal blockade, shock, and hyperkalemia] syndrome is a recently recognized clinical condition that may not always be promptly diagnosed. It is typically seen in elderly patients who have decreased renal function at baseline [1,2]. Joshua D. Farkas first described this clinical entity in 2016 when the combination of AV nodal blockade together with hyperkalemia led to profound bradycardia in a patient with underlying renal insufficiency [3]. Since then, several case reports have described the occurrence of this syndrome. Although numerous cases of the syndrome have been reported, there is significant variability in its precipitating factors and clinical presentation. We report a case of a patient on long-term amiodarone therapy who initially presented to the emergency department for gastrointestinal symptoms but was subsequently found to be in shock, which was deemed to have resulted from BRASH syndrome.

## Case presentation

The patient was a 97-year-old female with a medical history of heart failure with preserved ejection fraction, atrial fibrillation, hypertension, hyperlipidemia, and hypothyroidism who presented to the emergency department with a one-day history of diarrhea and vomiting. The patient’s home medications were as follows: amiodarone 200 mg daily, apixaban 2.5 mg daily, clopidogrel 75 mg daily, rosuvastatin 5 mg daily, losartan 25 mg daily, spironolactone 25 mg daily, and levothyroxine 50 mcg daily. The patient reported multiple episodes of non-bloody vomiting and diarrhea without fever, chills, or abdominal pain. There were no recent ill contacts, travel history, recent hospitalization, or recent antibiotics use, and no one else in the patient’s family had similar symptoms.

**Figure 1:**
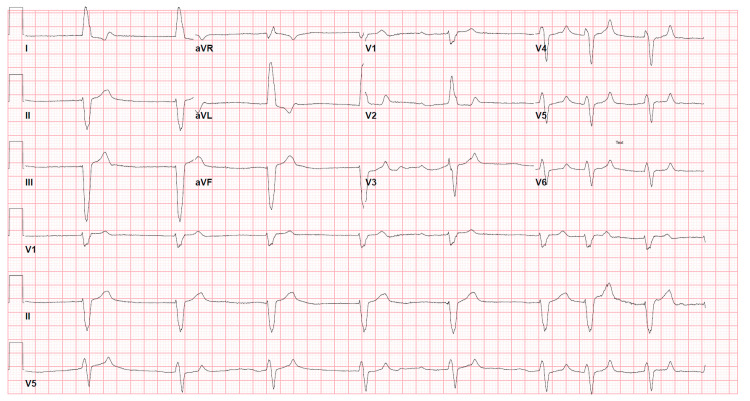
Admission EKG showing atrial fibrillation with a slow ventricular response and wide QRS complex EKG: electrocardiogram

Upon presentation, the patient was hypotensive (blood pressure of 90/40 mmHg) and bradycardic (heart rate of 51 beats/minute). Laboratory findings demonstrated a white blood cell count of 11.4 x 10^9^/L (no bands), serum potassium of 8.8 mEq/L, serum creatinine of 3.62 mg/dL (baseline serum creatine: 0.99 mg/dL), serum bicarbonate of 13 mEq/L, anion gap of 21, serum lactate of 2.8 mEq/L, and venous pH of 7.16. An initial EKG revealed atrial fibrillation with a slow ventricular response (heart rate of 51 beats/minute), left axis deviation, and wide QRS complex (QRS duration of 160 milliseconds) (Figure 1). It was determined that sepsis was not the cause of the patient's presentation, as the white blood cell count later decreased to 10.6 x 10^9^/L and, additionally, the blood cultures were negative throughout her hospital stay.

**Figure 2:**
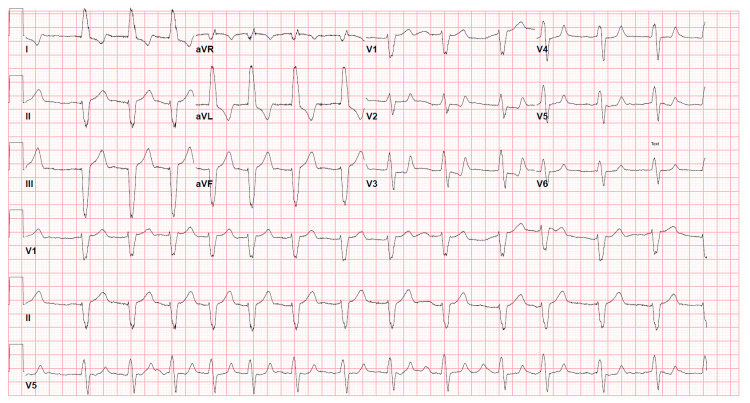
EKG obtained following 12 hours of the initiation of treatment showing improvement in bradycardia but the persistence of atrial fibrillation EKG: electrocardiogram

Losartan, spironolactone, and amiodarone were held due to hyperkalemia, hypotension, and bradycardia. The patient was given insulin with dextrose, intravenous calcium gluconate, albuterol nebulization, and polystyrene sulfonate to treat hyperkalemia. Intravenous fluid resuscitation was administered for hemodynamic support and treatment of acute renal failure. Sodium bicarbonate infusion was given to treat the acidosis. The patient was admitted to the ICU for close hemodynamic monitoring. Nephrology was consulted for possible renal replacement therapy, but the constellation of bradycardia, acute renal failure, hyperkalemia, anion gap metabolic acidosis, and hypotension improved after the above-mentioned treatment (Table 1). The repeat EKG and the cardiac monitor continued to show atrial fibrillation (Figure 2), and the heart rate remained stable in the range of 70-80 beats/minute on the first day of hospitalization. However, the patient was tachycardic on the second day of hospitalization with a resting heart rate in the range above 100. Because the patient’s shock was attributed in part to amiodarone, the decision was made not to restart the medication and instead to move towards rate control with metoprolol succinate 25 mg daily. On the day of discharge, the serum creatinine, potassium, bicarbonate, and anion gap returned to normal levels, and hence losartan and spironolactone were resumed. The patient was advised to continue to follow up with the cardiologist.

**Table 1:** Patient’s basic metabolic panel on admission and following the treatment

Variables	Day 1				Day 2	Day 3
	At presentation	2 hours post-treatment initiation	8 hours post-treatment initiation	12 hours post-treatment initiation		
Blood urea nitrogen (BUN) (mg/dL)	114	106	99	89	78	41
Serum creatinine (mg/dL)	3.62	3.48	2.81	2.30	1.81	1.19
Potassium (mEq/L)	8.8	7.3	5.8	4.4	4.4	4.2
Bicarbonate (mEq/L)	13	13	16	28	32	26
Anion gap	21	22	11	11	4	4

## Discussion

BRASH syndrome’s pathogenesis is characterized by a self-perpetuating cycle of bradycardia that is potentiated by the concomitant occurrence of medication use, hyperkalemia, and renal failure. This feedback loop is driven by the synergistic interplay between these factors, each of which contributes to and exacerbates the others in a self-reinforcing manner. Renal failure causes hyperkalemia and the potential accumulation of AV nodal blocking agents. The concomitant occurrence of hyperkalemia and the use of AV nodal blocking agents results in synergistic bradycardia, leading to a decrease in cardiac output and further diminishing renal perfusion and eventually worsening hyperkalemia, thereby perpetuating the vicious cycle of BRASH syndrome [1,2]. Hyperkalemia in BRASH syndrome is typically mild to moderate and the characteristic EKG changes of severe hyperkalemia (serum potassium level >7 mEq/L) such as widening of QRS complex may not be present [3].

The clinical presentation of BRASH syndrome can vary, with some cases demonstrating hypotension and hyperkalemia accompanied by acute kidney injury (AKI), while others show severe bradycardia necessitating transvenous pacing [2,4-6]. AV nodal blocking agents, e.g., beta-blockers (such as propranolol, atenolol, carvedilol, and metoprolol) and non-dihydropyridines calcium channel blockers (such as verapamil and diltiazem) are commonly implicated in BRASH syndrome [2,3]. While dihydropyridines calcium channel blockers (non-DHP CCBs) like amlodipine and nifedipine are typically not thought to be cardioactive, there have been cases of BRASH syndrome associated with non-DHP CCBs [7,8]. BRASH syndrome was diagnosed in our patient based on her clinical presentation of bradycardia, acute renal failure, hypotension, and hyperkalemia. We hypothesize that amiodarone, the sole AV nodal blocking agent in our case, could have contributed to the suppression of the AV node, leading to the emergence of BRASH syndrome in this patient. Prior case studies have also implicated amiodarone in the onset of BRASH syndrome [9,10]. The patient had been tolerating the above medications well for a long duration and the development of renal failure and subsequent risk of BRASH in this specific case was likely triggered by hypovolemia resulting from gastrointestinal losses.

BRASH syndrome can lead to multiorgan failure and understanding its pathophysiology is key to initiating appropriate management. Identifying the cycle and its different symptoms at the time of admission in patients with BRASH is crucial to reducing mortality as severe bradycardia can lead to cardiovascular collapse and severe hyperkalemia can lead to cardiac arrest [11,12]. The treatment should be targeted at correcting hyperkalemia and identifying the underlying cause while providing hemodynamic support for bradycardia and shock [3,12]. Stopping AV nodal blocking agents, correcting electrolytes, and fluid replacement are crucial steps in the treatment. Vasopressors, inotropes, and transvenous pacing may also be required in cases of refractory hemodynamic instability [3].

Due to the scarcity of cases, there are currently no guidelines available for the management of BRASH syndrome. In patients who develop BRASH syndrome, stopping the AV nodal blocking agents is recommended in the available literature but no specific time frame has been mentioned after which AV nodal blockers can be safely restarted. Moreover, the risk of restarting AV nodal blockers in these patient populations has not been established yet. Shared decision-making involving the patient and physician in terms of employing appropriate management needs to be made when reassessing the need to restart any other AV nodal blocking agents, especially in patients who need AV nodal blockade, as in our patient who required beta-blockers for heart rate control due to her underlying atrial fibrillation. This risk of developing BRASH in the future after resuming AV nodal blocking agents following its resolution remains high, especially in patients with decreased renal function at baseline [13]. It is therefore important to exercise caution when resuming agents like ACE-I and K-sparing diuretics too, as they can cause AKI and/or hyperkalemia, which can then intensify the effects of AV nodal blocking agents, causing bradycardia and potentially triggering BRASH syndrome. Therefore, frequent monitoring of serum electrolytes and creatinine through routine laboratory investigation and close follow-up visits with primary care physicians and/or cardiologists will be crucial in the early identification of acute renal failure or hyperkalemia, thus providing an opportunity to break the vicious cycle of BRASH syndrome that can possibly develop in these patients.

## Conclusions

BRASH syndrome is a rare but potentially life-threatening clinical entity. The primary contributing factor to this syndrome is the use of AV nodal blocking agents. Identifying the early signs and symptoms of BRASH syndrome is critical to reducing mortality. Further studies are necessary to establish guidelines for its management and the indications for the discontinuation and restarting of AV nodal blocking agents in patients with BRASH syndrome.
